# AKR1B10 promotes breast cancer metastasis through integrin α5/δ-catenin mediated FAK/Src/Rac1 signaling pathway

**DOI:** 10.18632/oncotarget.9672

**Published:** 2016-05-27

**Authors:** Chenfei Huang, Steven Verhulst, Yi Shen, Yiwen Bu, Yu Cao, Yingchun He, Yuhong Wang, Dan Huang, Chun Cai, Krishna Rao, Duan-Fang Liao, Junfei Jin, Deliang Cao

**Affiliations:** ^1^ Department of Medical Microbiology, Immunology & Cell Biology, Simmons Cancer Institute, Southern Illinois University School of Medicine, Springfield, IL 62794, USA; ^2^ Division of Stem Cell Regulation and Application, State Key Laboratory of Chinese Medicine Powder and Medicine Innovation in Hunan (incubation), Hunan University of Chinese Medicine, Changsha, Hunan 410208, China; ^3^ China-USA Lipids in Health and Disease Research Center, Guilin Medical University, Guilin, 541001, Guangxi, China

**Keywords:** AKR1B10, breast cancer metastasis, integrin α5, δ-catenin, Rac1

## Abstract

Aldo-keto reductase 1B10 (AKR1B10) is not expressed in normal breast, but upregulated in primary and metastatic breast cancers, being a negative prognostic factor. This study characterized the molecular mechanisms of AKR1B10-promoted breast cancer metastasis. Ectopic expression of AKR1B10 in breast cancer cells MCF-7 and MDA-MB-231 or siRNA-mediated silencing in BT-20 cells affected cell adhesion, migration and invasion in cell culture, and metastasis to the lung in the nude mice through upregulation of integrin α5 and δ-catenin. Silencing of integrin α5 or δ-catenin eradicated the cell adhesion and migration enhanced by AKR1B10, both of which acted synergistically. In these cells, the integrin α5 mediated focal adhesion kinase (FAK) signaling pathway was activated by AKR1B10, which, along with δ-catenin, stimulated Rac1-mediated cell migration and movement. In human primary and lymph node metastatic breast cancer, AKR1B10, integrin α5 and δ-catenin were correlatively upregulated with r=0.645 (p<0.0001) and r=0.796 (p<0.0001), respectively. These data suggest that AKR1B10 promotes breast cancer metastasis through activation of the integrin α5 and δ-catenin mediated FAK/Src/Rac1 signaling pathway.

## INTRODUCTION

Breast cancer (BC) accounts for approximately 40,000 deaths annually in the United States, and metastasis is the main cause of cancer mortality [1, 2]. Cancer metastasis is a multistep process that includes tumor cell adhesion to extracellular matrix (ECM), migration and invasion, intravasation into the circulatory system, and extravasation to distant tissues, eventually forming micrometastases [3]. The migration and invasion of tumor cells is utterly crucial in cancer metastasis. A tumor cell attaches to ECM and forms protrusions through focal adhesions (FA) at the leading edge; tractive force breaks the existing cell-ECM interactions at the trailing edge, allowing the cell to move forward [4]. Cell adhesion molecules (CAM) play a critical role in cell migration [5, 6]. Integrins, a major class of CAM, are important components of FA. The integrin family consists of 18α- and 8β-glycoprotein subunits, forming at least 25 distinct heterodimeric receptors. They specifically recognize and bind to specific ECM molecules and bi-directionally transport signals across the cell membrane, sensing environmental changes [7]. Integrins regulate cell adhesion and motility through a FA signaling cascade [8]. The integrin α/β clusters bind to ECM ligands, activating focal adhesion kinase (FAK). The activated FAK autophosphorylates its own tyrosine-397 residue and recruits Src family kinases that activate downstream signaling effectors, such as Rac1 [7]. Rac1 is a Rho GTPase that is located at leading edge of a moving cell and regulates Scar/WAVE-mediated actin polymerization and lamellipodia formation [9].

Integrin α5 interacts with integrin β1 to form a heterodimer, which functions with fibronectin as a ligand, regulating cell adhesion [10]. In human hepatocarcinoma cells, integrin α5 promotes cell adhesion and migration [11], and in breast cancer, integrin α5 is upregulated as an indicator of poor prognosis [12]. Blockage of integrin α5β1 function suppresses breast cancer cell proliferation and metastasis [13]. Delta-catenin is an armadillo protein that is primarily expressed in neurons, where it functions in cell morphogenesis and movement [14]. The δ-catenin is upregulated in breast, lung and brain cancers, promoting tumorigenesis and metastasis [15].

Aldo-keto reductase 1 B10 (AKR1B10) is a multifunctional protein, identified primarily from human hepatocellular carcinoma (HCC) [16]. AKR1B10 can efficiently converts cytotoxic and carcinogenic α, β-unsaturated carbonyl compounds into alcoholic forms with less toxicity, protecting the host cell from carbonyl damage [17–21]. AKR1B10 also promotes *de novo* fatty acid/lipid synthesis by stabilizing acetyl-CoA carboxylase-α (ACCA), a rate-limiting enzyme in the *de novo* fatty acid synthesis [22, 23]. In normal tissues, AKR1B10 is primarily expressed in the colon and small intestine and promotes epithelial cell proliferation regulating epithelial cell self-renewal [16, 24]. Targeted disruption of *AKR1B8* (an ortholog of *AKR1B10* in mouse) leads to diminished proliferation and migration of epithelial cells and increased susceptibility to carbonyl and oxidative stress-induced DNA damage and tumorigenesis [25]. In tumors, AKR1B10 is upregulated in breast, liver and lung cancers, promoting tumor growth and progression [26–29]. Overall, AKR1B10 functions as a protector of cells against carbonyl damage and a promoter of cell proliferation; but its role in tumorigenesis is tissue-context dependent. In breast cancer, AKR1B10 is also upregulated in the metastatic (78.0%) and recurrent (87.5%) tumors, indicating its potential role in breast cancer metastasis and recurrence [27]. This study clarified the molecular mechanism of action that AKR1B10 promotes breast cancer metastasis.

## RESULTS

### AKR1B10 promotes adhesion, migration and invasion of breast cancer cells

**Figure 1:**
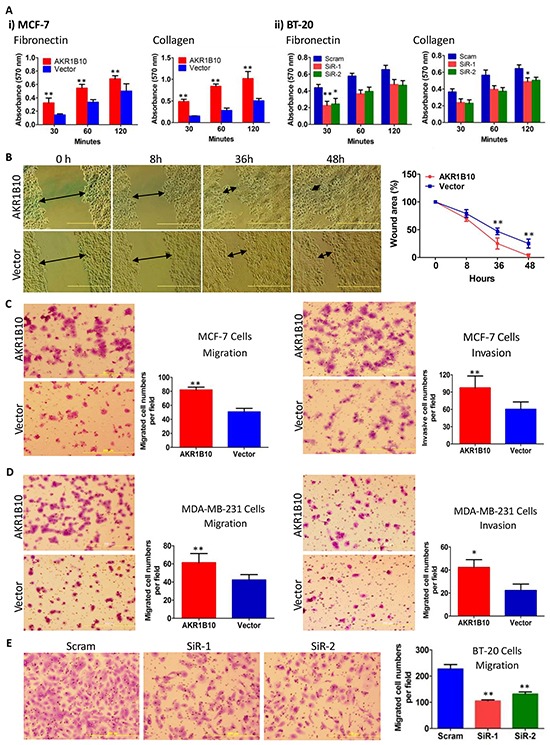
AKR1B10 increases adhesion, migration and invasion of breast cancer cells AKR1B10 was ectopically expressed in MCF-7 and MDA-MB-231 cells, or silenced in BT-20 cells. **A.** Cell adhesion to fibronectin or collagen-coated plates. **B.** Wound-healing of MCF-7 cells. Scale bar = 500μm. **C.** Transwell migration (*left*) and Boyden chamber invasion (*right*) of MCF-7 cells. **D.** Transwell migration (*left*) and Boyden chamber invasion (*right*) of MDA-MB-231 cells. **E.** Transwell migration of BT-20 cells. All data represent mean ± SD from three independent experiments. *, p<0.05 and **, p<0.01 compared to the vector control. Scram, scrambled siRNA; siR-1, AKR1B10 siRNA-1; and siR-2, AKR1B10 siRNA-2.

AKR1B10 is upregulated in human breast cancer and correlates with lymph node metastasis [27]. The current study demonstrated that ectopic expression of AKR1B10 in breast cancer cells MCF-7 (Supplementary Figure S1A) enhanced cell adhesion to fibronectin or collagen-coated plates (Figure 1A, *left panel*), but not to gelatin or laminin-coated plates (Supplementary Figure S2A). Wound-healing assays showed that MCF-7 cells with AKR1B10 expression migrated into and fully covered the wound areas within 48 hours in the fibronectin-coated plates, but vector control cells did not (Figure 1B). Boyden chamber assays showed that AKR1B10 expression enhanced the transmigration of MCF-7 cells through fibronectin-coated membrane (Figure 1C, *left*) and invasion in Matrigel (Figure 1C, *right*) when compared to vector control cells. Similarly, ectopic expression of AKR1B10 in MDA-MB-231 cells (Supplementary Figure S1B) also enhanced their migration and invasion (Figure 1D). In contrast, silencing of AKR1B10 in breast cancer cells BT-20 (Supplementary Figure S1C) markedly lowered down their adhesion to fibronectin or collagen-coated plates (Figure 1A, *right panel*), but not to laminin or gelatin-coated plates (Supplementary Figure S2B). The transmigration of BT-20 cells with silencing of AKR1B10 was decreased by 40-50% (Figure 1E). These data suggest that AKR1B10 promotes adhesion, migration and invasion of breast cancer cells.

### AKR1B10 upregulates the expression of integrin α5 and δ-catenin in breast cancer cells

**Figure 2:**
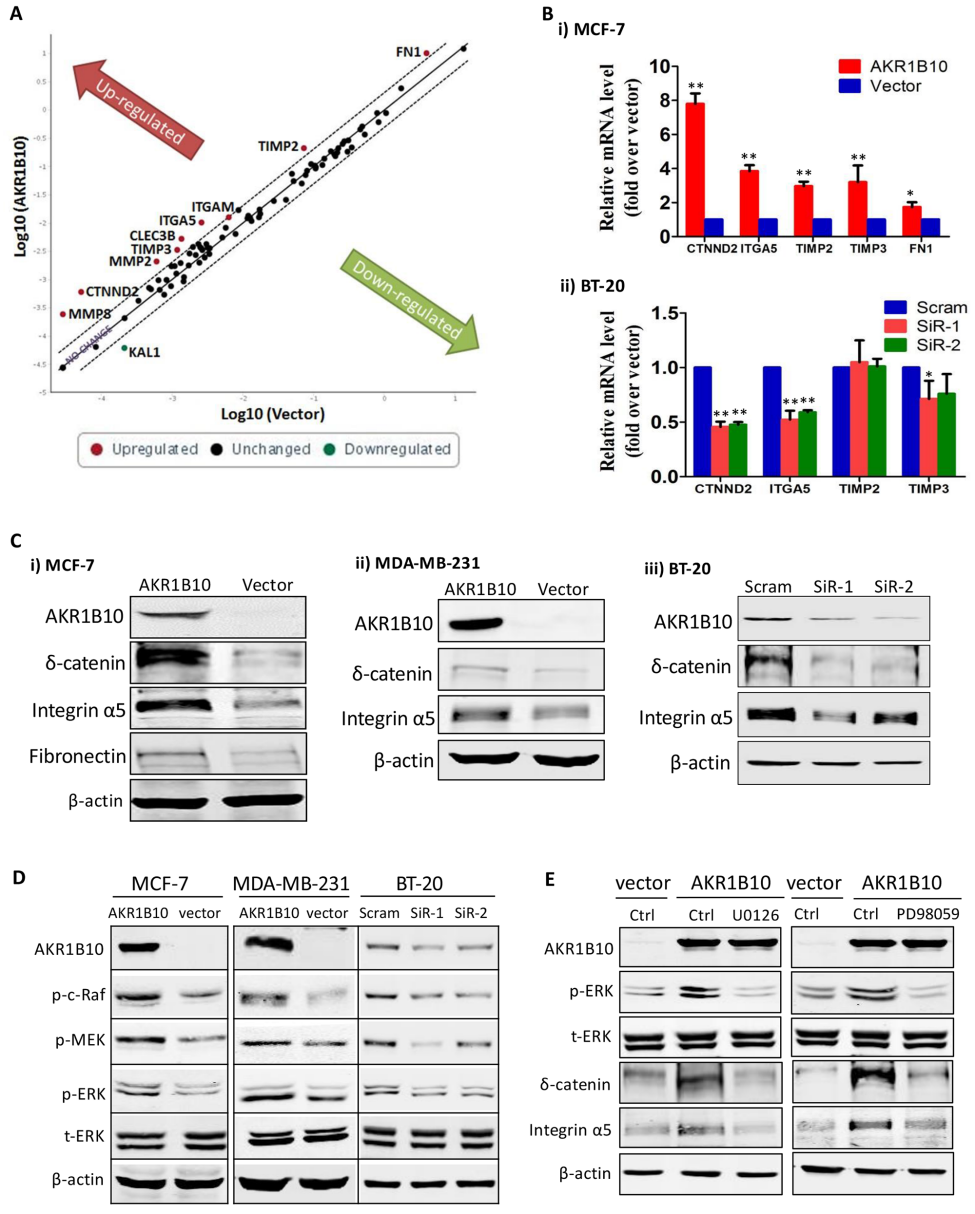
AKR1B10 upregulates integrin α5 and δ-catenin in breast cancer cells **A.** RT profiler PCR array results, showing expression of 84 cell adhesion molecules and extracellular proteins in MCF-7 cells with ectopic expression of AKR1B10. **B.** qRT-PCR and **C.** Western blot, confirming expression of interest genes in MCF-7, MDA-MB-231 and BT-20 cells. **D.** ERK signaling activation, showing p-Raf, p-MEK and p-ERK in MCF-7, MDA-MB-231 and BT-20 cells. **E.** Inhibition of integrin α5 and δ-catenin expression by MEK1/2 inhibitors, PD98059 (10μM) and U0126 (10μM), showing expression of p-ERK1/2, integrin α5, and δ-catenin in MCF-7 with ectopic AKR1B10 expression.

Cell movement is mediated by cell-matrix interactions; and CAMs play a critical role in this process [6]. Therefore, we explored the potential effects of AKR1B10 on the expression of CAMs and ECMs in MCF-7 cells using an Adhesion-RT^2^ Profiler PCR array that allows for a quantitative assessment of 84 CAM and ECM genes (Figure 2A and Supplementary Figure S3). Using qRT-PCR and Western blot, we further confirmed the upregulation of CTNND2 (δ-catenin), ITGA5 (integrin α5) and FN1 (fibronectin) by AKR1B10 at both mRNA (Figure 2B) and protein levels (Figure 2C) in MCF-7 cells. Consistently, both δ-catenin and integrin α5 were also upregulated by AKR1B10 in MDA-MB-231 cells (Figure 2C, *middle*). In contrast, silencing of AKR1B10 in the BT-20 cells lowered down mRNA (Figure 2B) and protein (Figure 2C) levels of δ-catenin and integrin α5. Of note, the effects of AKR1B10 on CAM expression displayed a cellular difference. FN1, CLEC3B, MMP2, MMP8, TIMP2 and TIMP3 were upregulated in MCF-7 cells by AKR1B10, but not notably affected in BT-20 cells by AKR1B10 silencing, suggesting a cell-context dependence. Nevertheless, AKR1B10 consistently upregulates the expression of δ-catenin and integrin α5 in all tested breast cancer cells.

We further asked how AKR1B10 upregulates integrin α5 and δ-catenin expression. The ERK signaling is a critical participant of focal adhesion assembly and disassembly [30]. We thus examined the activity of ERK signaling cascade and the effects on the expression of integrin α5 and δ-catenin. As shown in Figure 2D, the phosphorylated c-Raf, MEK1/2 and ERK1/2 were increased in the MCF-7 and MDA-MB-231 cells with AKR1B10 expression, but decreased in the BT-20 cells with AKR1B10 silencing. Pharmacological inhibition of ERK signaling by MEK1 inhibitors PD98059 (10 μM) and U0126 (10 μM) attenuated the expression of integrin α5 and δ-catenin upregulated by AKR1B10 (Figure 2E). These data suggest that AKR1B10 regulates the expression of integrin α5 and δ-catenin through the c-Raf/MEK/ERK signaling pathway.

### Integrin α5 and δ–catenin mediate cell adhesion and migration through focal adhesion signaling cascade

**Figure 3:**
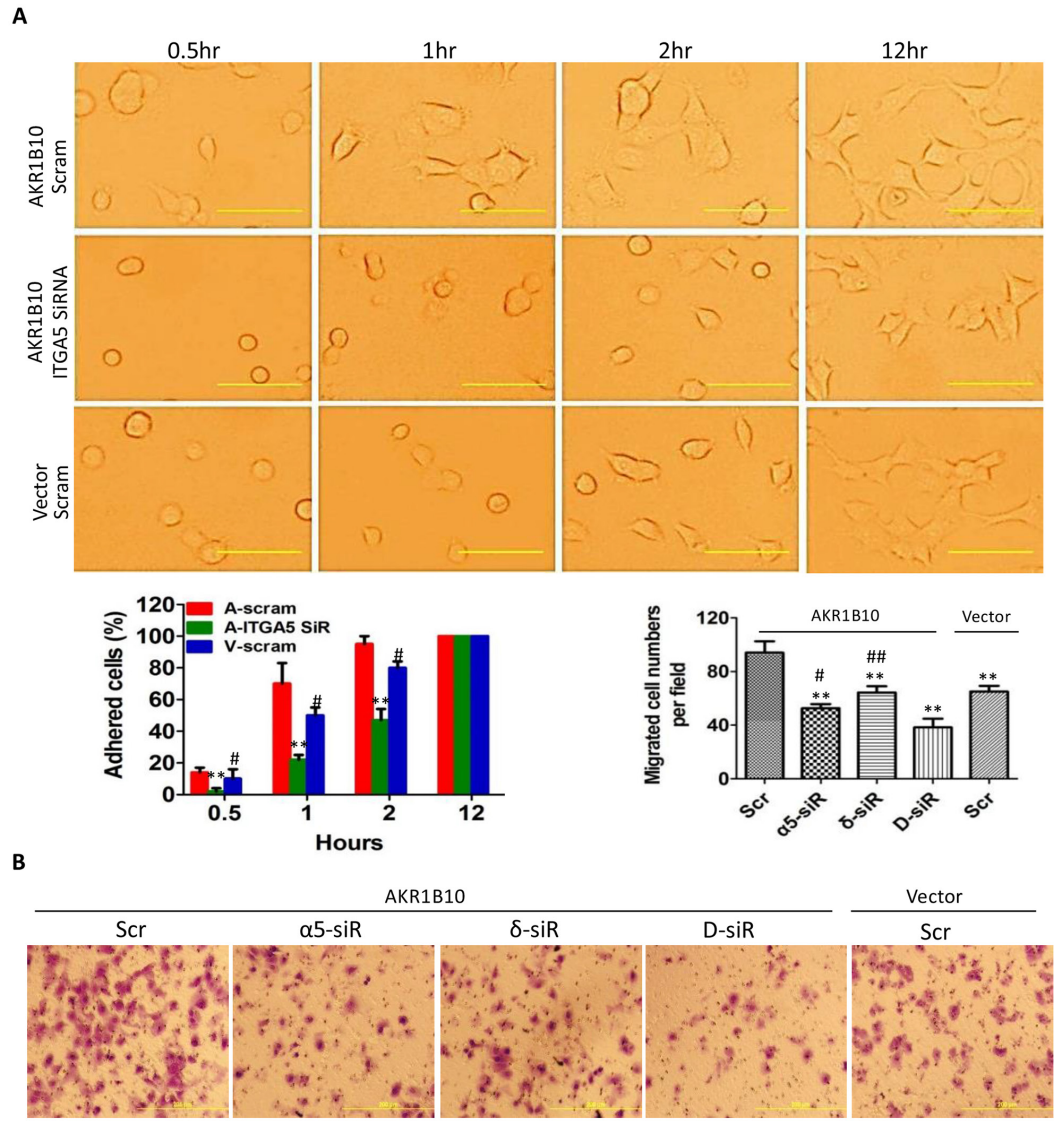
Integrin α5 and δ-catenin mediate the AKR1B10-promoted cell adhesion and migration **A.** Cell adhesion. *Middle left:* quantitation expressed as percentage of adhered cells at each time point over the cells adhered at 12 hours. Scale bar = 25μm. Data represent mean ± SD from three independent assays. **, p<0.01, compared to A-Scram; #, p<0.05 and ##, p<0.01, compared to A-Scram. A-Scram, AKR1B10 expression MCF-7 cells treated with scramble siRNA; V-Scram, vector control MCF-7 cells treated with scramble siRNA; and A-ITGA5 SiR, AKR1B10 expression MCF-7 cells treated with integrin α5 siRNA. **B.** Transwell migration of MCF-7 cells with silencing of integrin α5, δ-catenin, or both. Data represent mean ± SD from three independent assays. *Middle right:* quantitation of migrated cells. Data represent mean ± SD from three independent experiments. **, p<0.01 compared to A-Scr control; #, p<0.05 and ##, p<0.01 when compared to D-siR. Scr, scrambled siRNA; α5-siR, integrin α5 siRNA; δ-siR, δ-catenin siRNA; and D-siR, double (integrin α5 plus δ-catenin) siRNA.

We further assessed the role of integrin α5 and δ-catenin in the AKR1B10-enhanced adhesion and migration of breast cancer cells by gene silencing (Supplementary Figure S4). Our results showed that silencing of integrin α5 by siRNA in MCF-7 cells attenuated the AKR1B10-enhanced cell adhesion to fibronectin-coated plates (Figure 3A) and the transmigration in Boyden chambers (Figure 3B). Silencing of δ-catenin also lowered down cell transmigration, but to a less content; and double silencing of both integrin α5 and δ-catenin demonstrated a synergistic role in suppression of the cell transmigration (Figure 3B). These data suggest that AKR1B10 promotes the adhesion and migration of breast cancer cells through the integrin α5 and δ-catenin mediated mechanism. The integrin α5 and δ-catenin function synergistically in this process.

**Figure 4:**
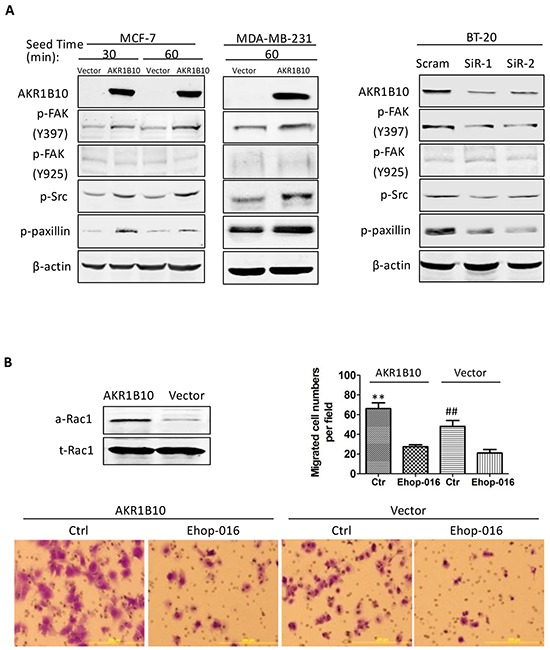
AKR1B10 activates the focal adhesion-Rac1 signaling cascade **A.** Activation of focal adhesion signaling cascade, showing p-FAK397, p-FAK925, p-Src and p-paxillin levels in indicated cells. Scram, scrambled siRNA; siR-1, AKR1B10 siRNA-1; siR-2, AKR1B10 siRNA-2. **B.** Rac1-mediated cell migration. *Upper left*: Active Rac1 (a-Rac1) examined by pull-down assays with PAK-PBD beads. Active Rac1 pulled down and total Rac1 (t-Rac1) in cell extracts were assessed by Western blot. *Lower panel images*: Effects of Rac1 inhibitor, EHop-016, on MCF-7 cell Transwell migration. *Upper right:* Quantitation of migrated cells. Data denote mean ± SD from three independent experiments. ** p<0.01, compared to EHop-016-treated MCF-7 cells with AKR1B10 expression or with a vector control. ## p<0.01 compared to EHop-016-treated MCF-7 cells with AKR1B10 expression or with a vector control.

Integrins interact with ECM proteins and activate a focal adhesion-mediated signaling cascade to drive cell movement. This process involves the phosphorylation and activation of FAK, Src, paxillin and Rac1 [7]. We estimated the effects of AKR1B10 expression on the focal adhesion signaling cascade. As shown in Figure 4A (*left panel*), ectopic expression of AKR1B10 in the MCF-7 and MDA-MB-231cells enhanced the phospho-Y397FAK level, but not the phospho-Y925/FAK. The Y397-phosphorylated FAK provides a platform for Src association and phosphorylation, which in turn activates downstream effectors Y118-paxillin (Figure 4A, *left panel*). In contrast, AKR1B10 silencing in BT-20 cells led to decrease in phospho-Y397 FAK, phospho-Src and phospho-paxillin (Figure 4A, *right panel*). As a downstream effector of the FAK/Src/paxillin signaling, Rac1 promotes cell motility through cytoskeleton remodeling [31]. Using pull-down assays, we found that the Rac1 was activated in MCF-7 cells (Figure 4B, *upper left*). Pharmacological inhibition of the Rac1 activity by Ehop-016 attenuated the cell migration increased by AKR1B10 (Figure 4B). These data suggest that AKR1B10 promotes cell migration via the integrin α5 mediated focal adhesion signaling cascade. The Rac1 functions as a downstream effector in this process.

### AKR1B10 promotes lung metastasis of MDA-MB-231 cells

**Figure 5:**
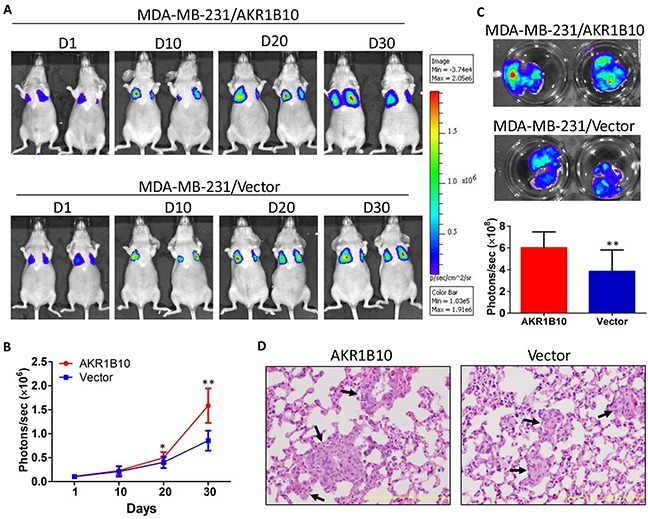
AKR1B10 promotes the lung metastasis of MDA-MB-231 cells in female nude mice Mice (n=5 per group) wereinjected via tail vein with MDA-MB-231 cells (1×10^6^) labeled with luciferase. **A.** Bioluminescent images of representative mice at days 1, 10, 20 and 30 post tail vein injection. **B.** Quantification of bioluminescent strengths at photon/second. **C.** *Ex vivo* imaging of lungs. At the end, mice were sacrificed and the lungs were excised for *ex vivo* bioluminescent imaging. **D.** H&E histology of lung metastatic tumors (arrows). Scale bar = 200μm.

To confirm the *in vitro* data that AKR1B10 promotes migration and invasion of breast cancer cells, we extended this study to animals. As shown in Figure 5, AKR1B10 promoted the lung metastasis of MDA-MB-231 cells as measured by the *in vivo* and lung *ex vivo* bioluminescent imaging. At the endpoint, mice were euthanized and the lungs were excised for histological analysis; and results showed that metastatic nodules were formed in lungs and larger in the MDA-MB-231 cells with AKR1B10 expression when compared to vector control cells. These data suggest that AKR1B10 promotes the lung metastasis and growth of breast cancer cells.

### AKR1B10, integrin α5, and δ-catenin are correlatively upregulated in human primary and metastatic breast cancers

**Figure 6:**
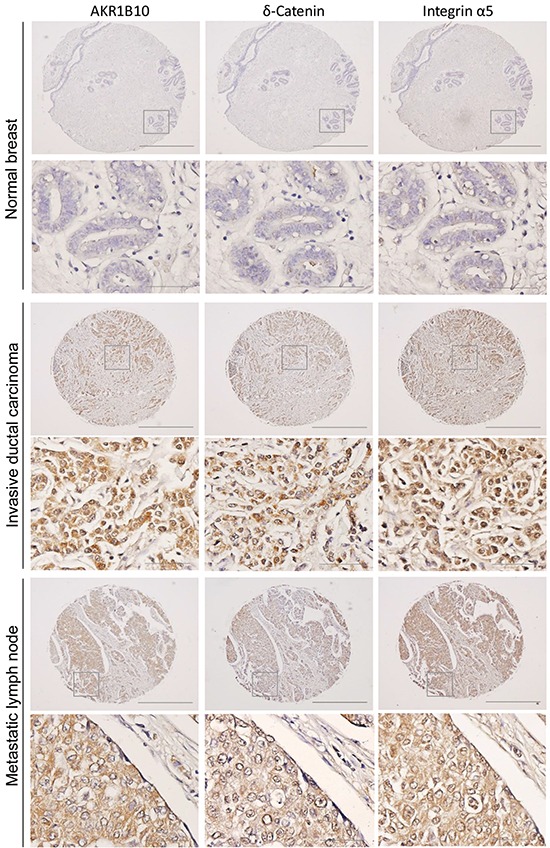
AKR1B10, integrin α5 and δ-catenin are correlatively upregulated in human breast cancer Adjacent tissue microarrays were stained by immunohistochemistry for the expression of AKR1B10, integrin α5 and δ-catenin in normal breast, primary breast cancer, and lymph node metastatic tumors. The images show representative tissues with these three protein expression. Scale bar = 50μM (40×) or 500μM (4×).

To evaluate translational relevance of the study results, we further investigated the expression and correlations of AKR1B10, integrin α5 and δ-catenin in human breast cancer tissues using adjacent sections of tissue microarrays. As shown in Figure 6 and Supplementary Figure S5, AKR1B10, integrin α5 and δ-catenin were upregulated in breast cancers, particularly in the metastatic lymph nodes. Correlative analyses using Spearman correlation indicated that the expression of AKR1B10, integrin α5 and δ-catenin was correlated to each other (Table 1); multivariate canonical correlations of AKR1B10, integrin α5 and δ-catenin expression were r=0.645 (p<0.0001) in primary breast cancer and r=0.796 (p<0.0001) in metastatic lymph nodes.

**Table 1:** Expression and correlation of AKR1B10, integrin α5, and δ-catenin in primary and metastatic breast cancers

	**Invasive ductal carcinomas (N=50)**				**Metastatic lymph nodes (N=40)**			
**Expression levels**								
**Intensity**	**0 (%)**	**1 (%)**	**2 (%)**	**3 (%)**	**0 (%)**	**1 (%)**	**2 (%)**	**3 (%)**
**AKR1B10**	7 (14.0)	27 (54.0)	11 (22.0)	5 (10.0)	6 (15.0)	18 (45.0)	5 (12.5)	11 (27.5)
**δ-Catenin**	10 (20.0)	27 (54.0)	8 (16.0)	5 (10.0)	7 (17.5)	18 (45.0)	5 (12.5)	10 (25.0)
**Integrin α5**	12 (24.0)	30 (60.0)	5 (10.0)	3 (6.0)	3 (7.5)	23 (57.5)	6 (15.0)	5 (20.0)
**Correlation**								
	**AKR1B10**	**δ-Catenin**	**Integrin α5**			**AKR1B10**	**δ-Catenin**	**Integrin α5**
**AKR1B10**		r=0.58785 p<0.0001	r=0.54985 p<0.0001	**AKR1B10**			r=0.69531 p<0.0001	r=0.74737 p<0.0001
**δ-Catenin**			r=0.55669 p<0.0001	**δ-Catenin**				r=0.65259 p<0.0001

## DISCUSSION

AKR1B10 is upregulated in multiple human tumors, including liver, lung, and breast cancers, functioning as a potential negative prognostic factor. However, little is known of the molecular mechanisms of action. This study demonstrated for the first time that AKR1B10 promoted breast cancer metastasis through activation of the integrin α5 and δ-catenin mediated FAK/Src/Rac1 signaling pathway. This mechanism was dissected via *in vitro* cellular studies and confirmed in *in vivo* metastatic animal models and *ex vivo* human tissues. This discovery defines AKR1B10 as a new oncogenic factor in the growth and progression of breast cancer.

Ectopic expression of AKR1B10 in breast cancer cells promoted their adhesion to fibronectin and collagen, migration in fibronectin-coated plates and invasion in Matrigel. These are in consistence with the clinical settings where AKR1B10 expression correlates with lymph node metastasis and worse disease-free survival [27]. Cell interaction and movement in the microenvironment are regulated by CAMs. AKR1B10 upregulated integrin α5, δ-catenin and fibronectin in breast cancer cells. Integrin α5 is a membrane receptor with fibronectin as a ligand, playing a vital role in adhesion and migration of cancer cells [32]; δ-Catenin is upregulated in human cancers and promotes tumor metastasis [15, 33]. This study found that in breast cancer cells, silencing of integrin α5 or δ-catenin alone eradicated the cell adhesion and migration enhanced by AKR1B10; and co-silencing of both integrin α5 and δ-catenin displayed a synergistic effect, suggesting that the integrin α5 and δ-catenin both function as the downstream effectors of AKR1B10 in regulation of the adhesion and migration of breast cancer cells.

How do integrin α5 and δ-catenin mediate the AKR1B10-promoted adhesion and migration of breast cancer cells then? Integrin α5 associates with integrin β1 to form an integrin α5β1 cluster, which recognizes and binds to extracellular fibronectin. The integrin α5β1–fibronectin complex triggers FA formation and activates focal adhesion kinase (FAK) mediated-signaling pathway [11, 34]. In this pathway, the activated FAK autophosphorylates its tyrosine-397 residue to recruit and phosphorylate Src kinase, which in turn activates the downstream signaling cascades, such as paxillin-Rac1 and FAK (pY925)-ERK1/2 [7]. This study showed an increase of phospho-Y397/FAK, phospho-Src, and phospho-paxillin levels and Rac1 activity, but the phospho-Y925/FAK was not altered, suggesting that the integrin α5β1–fibronectin complex promotes the adhesion and migration of breast cancer cells through the FA-mediated FAK (pY397)/Src/paxillin/Rac1 signaling pathway, rather than the FAK (pY925)-ERK1/2 cascade. Delta-catenin also regulates the activity of Rho GTPases (e.g., RhoA, Rac1 and Cdc42), promoting lamellipodia protrusion and cell mobility [33, 35]. The synergistic role of integrin α5 and δ-catenin in the AKR1B10-promoted adhesion and migration of breast cancer cells suggests that Rac1 is a common effector of these two CAMs. To be noted, δ-catenin also binds to E-cadherin and promote cell migration [15]. Our data showed that AKR1B10 did not notably affect the expression, phosphorylation or subcellular distribution of E-cadherin in MCF-7 cells (Supplementary Figure S6), suggesting that δ-catenin acts through the Rac1 rather than E-cadherin signaling.

We further asked how AKR1B10 upregulates integrin α5 and δ-catenin expression. The ERK signaling regulates focal adhesion assembly and disassembly [30]. In this study, we found that the c-Raf/MEK/ERK signaling cascade was activated by AKR1B10, which may be responsible for upregulation of integrin α5 and δ-catenin as the pharmacological inhibition by MEK1 inhibitors PD98059 (10μM) and U0126 (10μM) attenuated integrin α5 and δ-catenin expression upregulated by AKR1B10. We do not have yet a direct explanation of how the ERK signaling is activated in these cells. In the FAK-mediated focal adhesion signaling, the phosphorylation of FAK at Y925 could activate the ERK signaling [7], but in the tested cells, the phospho-Y925/FAK level was not altered. In contrast, the phosphorylation level of C-Raf and MEK1/2 was increased, suggesting that the ERK signaling may be activated through a G-protein coupled membrane receptor mechanism. Further study is warranted.

The promoting role of AKR1B10 in breast cancer metastasis was further confirmed in *in vivo* animal modeling and *ex vivo* clinical samples. In the female nude mice, AKR1B10 enhanced lung metastasis and nodular growth of MDA-MB-231 cells, demonstrating large metastatic nodules. In clinical specimens of breast cancer, we observed a high correlation in expression between AKR1B10 and integrin α5 and δ-catenin, being supportive to our mechanistic studies in cell culture. Of note, AKR1B10 promoted the adhesion, migration and invasion of MCF-7 cells *in vitro*, but AKR1B10 alone could not convert the non-invasive MCF-7 cells into invasive, lung metastatic cells (data not shown), suggesting that AKR1B10 may function as a promoter, but not an initiator, of breast cancer metastasis.

**Figure 7:**
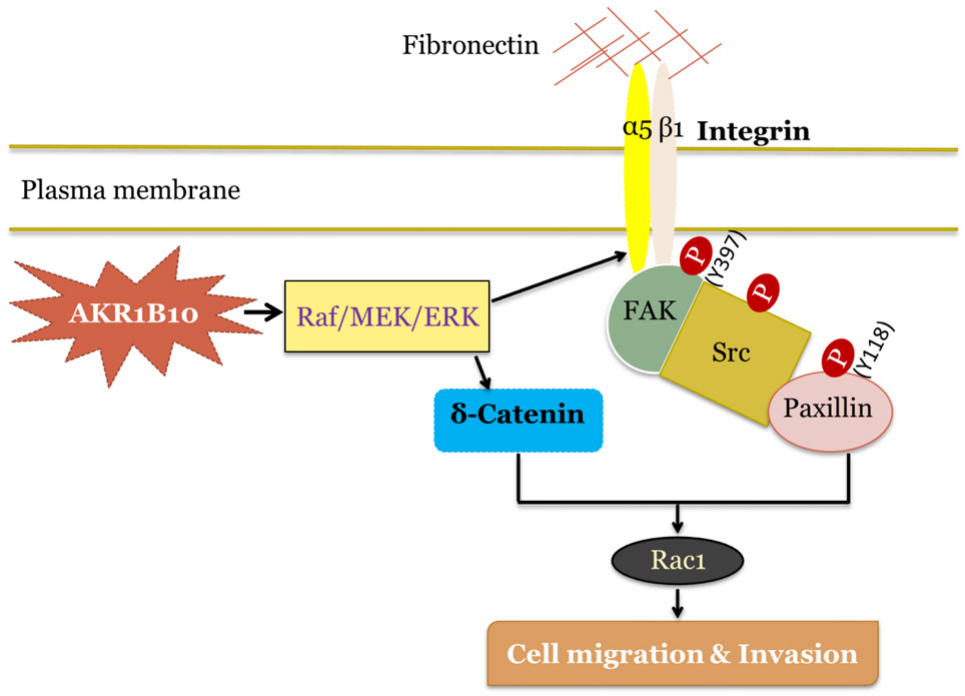
Hypothetic model of AKR1B10 in breast cancer metastasis AKR1B10 activates Raf/MEK/ERK signaling cascade, which upregulates integrin α5. Integrin α5 associates with integrin β1, binds to fibronectin and activates focal adhesion signaling by phosphorylation of focal adhesion kinase (FAK) at residue Y397. Activated FAK provides a platform for Src binding and phosphorylation, which is followed by the activation of Y118-paxillin and Rac1. Meanwhile, AKR1B10-activated Raf/MEK/ERK signaling increases δ-catenin level, which in turn activates Rac1, and synergistically promotes breast cancer cell migration and invasion.

In summary, this study demonstrated that AKR1B10 promotes breast cancer metastasis at the levels of *in vitro* cell culture, *in vivo* animal and *ex vivo* clinical settings. This study also demonstrated that AKR1B10 promotes breast cancer metastasis through activation of the integrin α5 and δ-catenin mediated FAK/Src/Rac1 signaling cascade, in which the integrin α5 and δ-catenin function synergistically (Figure 7). Our results suggest that AKR1B10 is a new oncogenic factor in breast cancer metastasis and a potential target for metastatic intervention.

## MATERIALS AND METHODS

### Cell culture

MCF-7, BT-20, MDA-MB-231, and 293T cells purchased from American Type Culture Collection (ATCC, VA) were maintained in indicated medium at 37°C, 5% CO_2_. For 2D culture, cells were seeded at 200 cells per 60-mm culture dish and incubated in indicated medium for 14 days; colonies were fixed by methanol (cooled at −20°C) for 10 min and visualized by 0.1% crystal violet. Plating efficiency was calculated as: Colony number/seeded cell number. The 3D culture was done in growth factor-reduced Matrigel (BD Biosciences, CA) [36]. Cells (4000/well) were seeded. Acini were photographed by a phase contrast microscopy (Carl Zeiss, CA).

### AKR1B10 ectopic expression

The full-length AKR1B10 cDNA [16] was inserted into pCDH lentiviral expression vector with a GFP reporter (System Biosciences, CA). After packaging in 293T cells, AKR1B10 and empty pCDH lentiviral particles were introduced into cells with standard procedures. GFP-labeled cells were sorted for a homogeneous population.

### AKR1B10, integrin α5 and δ-catenin silencing

AKR1B10 siRNA previously characterized [20] and integrin α5 and δ-catenin siRNAs (Santa Cruz Biotechnology, CA.) were introduced into cells with scrambled siRNA as a control. Silencing efficiency was evaluated by Western blot analysis.

### qRT-PCR

Total RNA was extracted using Trizol® reagent (Invitrogen, CA) and quantitated at OD_260_. Total RNA (1.0μg) was treated with RNase-free DNase 1 and reverse-transcribed into cDNA with random primers and Superscript II®retrotranscriptase (Invitrogen, CA). qRT-PCR was run in a mix of 2.0μl cDNA, 10 μmol/L primers and 2×SYBR Green FastMix (Quanta Biosciences, CA). Relative expression levels were analyzed by comparative C_T_ (ΔΔC_T_) with GAPDH as an internal control. Gene specific primer sequences for qRT-PCR were shown in Supplementary Table S1.

### RT^2^ Profiler PCR Array

Human Extracellular Matrix & Adhesion Molecules RT^2^ Profiler™ PCR Array (Qiagen, CA) was used for expression profiling analysis of cell adhesion molecules (CAM). This array simultaneously profiled 84 CAM genes with five housekeeping genes for data normalization.

### Western blot

Protein lysates, SDS-PAGE, and Western blotting was performed as previously described [37].

### Cell adhesion assays

The 96-well plates (Thermo Scientific, IL) were coated with 100μl of 10 μg/ml fibronectin, collagen, laminin or gelatin (BD Biosciences, CA) at 37°C for 1 hour, followed by blocking with 1% BSA for 45 minutes. Serum-starved cells were suspended with 2.0mM EDTA, and spread at 5×10^4^ cells per well in 100μl medium containing 1% FBS. After incubation for 30, 60 or 120 minutes at 37°C, 5% CO_2_, attached cells were fixed with 4% paraformaldehyde and stained with 0.2% crystal violet in 10% ethanol for quantitation at 570 nm using a Multiscan Spectrum reader (Thermo Scientific, IL).

### Cell migration, invasion and wound-healing assays

Migration assays were performed using Boyden chamber Transwells with 8μm pore size membrane (Costar, MA) that were coated with 10μg/ml fibronectin in PBS at 4°C overnight. Cells (5×10^4^-8×10^4^/well) were placed in the upper chamber. After 24 hours, cells in the inner surface of inserts were removed by cotton swabs and the cells on the outer surface of the inserts were fixed, stained and counted under a microscope (Olympus, Japan). Cell invasion was assayed in matrigel-coated Boyden chambers. Cells (5×10^4^/well) were added onto the top of chamber. After 48 hours, cells were fixed, stained and counted. For Rac1 inhibition, cells were exposed to a Rac1 inhibitor, Ehop-016 (Sigma, MO) at 2μM for 24 hours and then seeded into Boyden chamber. For wound-healing assays, a linear scratch was created in monolayer cells using a 200μl pipette tip and the healing was monitored under a microscopy (Olympus, Japan).

### Rac1 activity assay

Rac1 activity was measured by a Rac1 assay kit (Cytoskeleton, CO) according to the manufacturer protocol. Cells were allowed to adhere to fibronectin-coated plates for 60 minutes in the medium containing 1% FBS before assays.

### Lung metastasis

The luciferase-labeled MDA-MB-231 cells (1×10^6^ in 100μl of PBS) that express AKR1B10 or vector control were injected into the tail vein of 5-week-old female BALB/c nude mice. *In vivo* lung bioluminescent imaging was conducted using an IVIS-200 Imaging System (Caliper Life Sciences, MA) to monitor metastatic tumors for 5 weeks. Bioluminescence was quantified as total photon/s using Living Image software (Caliper Life Sciences, MA). For *ex vivo* lung imaging, 200 μl of D-luciferin (150 mg/kg in PBS) was injected intraperitoneally, and mice were euthanized. Lungs were excised and imaged for 1-3 seconds in 12-well plates containing 300μg/ml D-luciferin in PBS [38]. Thereafter, the lungs were sliced and fixed in 10% formalin for histology.

### Tissue microarrays

Adjacent breast cancer tissue microarrays containing 50 invasive carcinomas, 40 lymph node metastatic tumors and 10 adjacent normal tissues were purchased from US Biomax (Rockville, MD). Standard immunohistochemistry [25] was performed for AKR1B10, integrin α5, or δ-catenin expression.

### Statistical analysis

Statistical analyses were performed using Student`s *t* tests with INSTAT statistical analysis package (Graph Pad software, CA). Significance was defined as p<0.05. Correlation analyses were conducted using Spearman correlation and multivariate canonical correlation.

## SUPPLEMENTARY FIGURES AND TABLES




