# Supplementary material for: AKR1B10 promotes breast cancer metastasis through integrin α5/δ-catenin mediated FAK/Src/Rac1 signaling pathway
Source: Oncotarget. 2016 May 27;7(28):43779–91. doi: 10.18632/oncotarget.9672 (PMC5190059; doi:10.18632/oncotarget.9672)
Supplement: Supplementary file 1 [file oncotarget-07-43779-s001.pdf]

SUPPLEMENTARY FIGURES AND TABLE

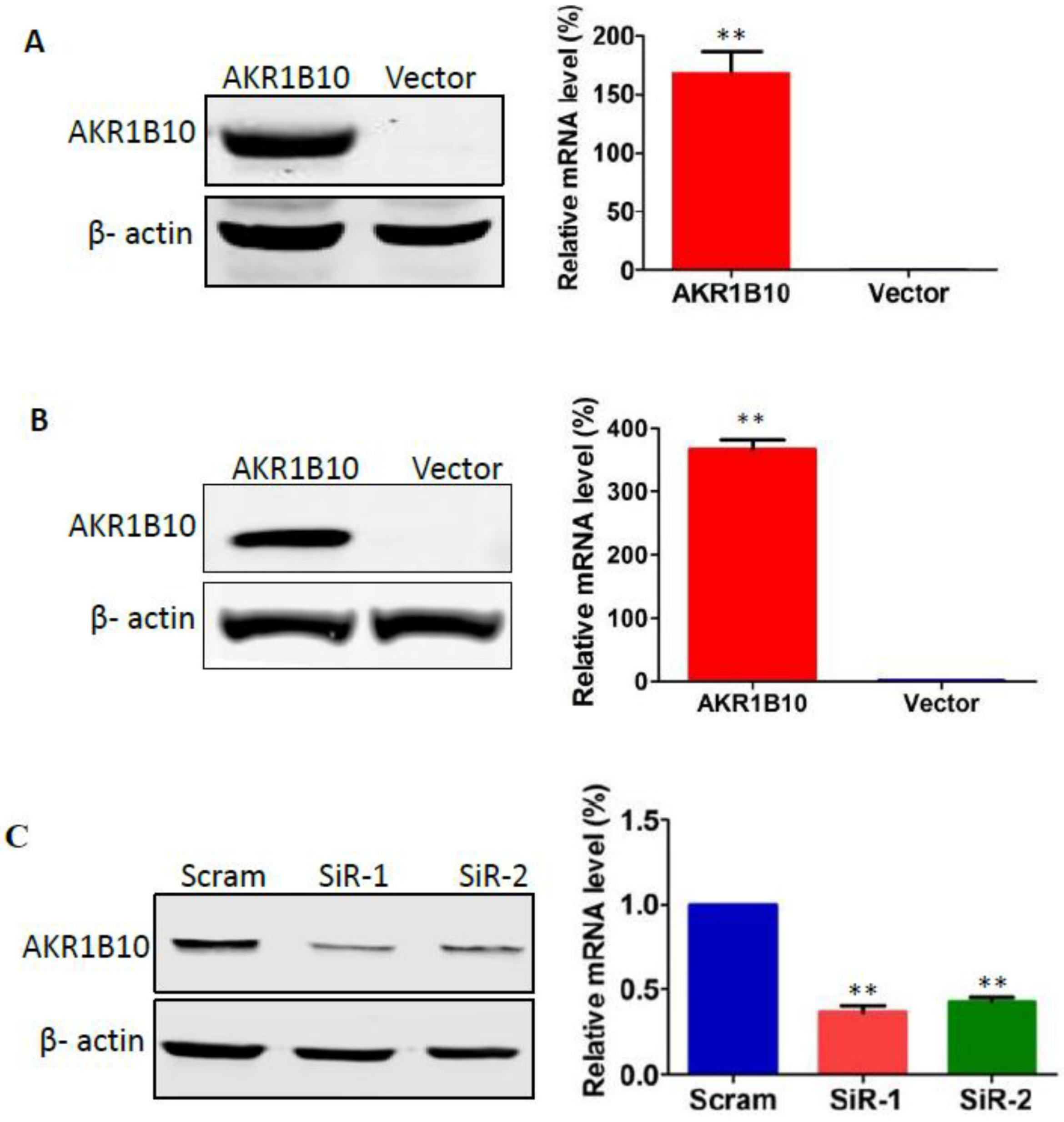

Supplementary Figure S1: Targeted expression of AKR1B10 in three breast cancer cell lines. A. Ectopic expression of AKR1B10in MCF-7 cells. *Left:*AKR1B10 protein by Western blot; *Right:*AKR1B10 mRNA by qRT-PCR. **B.** Ectopic expression of AKR1B10in MDA-MB-231 cells. *Left:*AKR1B10 protein by Western blot; *Right:*AKR1B10 mRNA by qRT-PCR. **C.** AKR1B10 silencing in BT-20 cells. *Left:*AKR1B10 protein by Western blot; *Right:*AKR1B10 mRNA by qRT-PCR. Scram, scrambled siRNA; SiR-1, AKR1B10 siRNA-1; and SiR-2, AKR1B10 siRNA-2. GAPDH mRNA was used as an internal control.

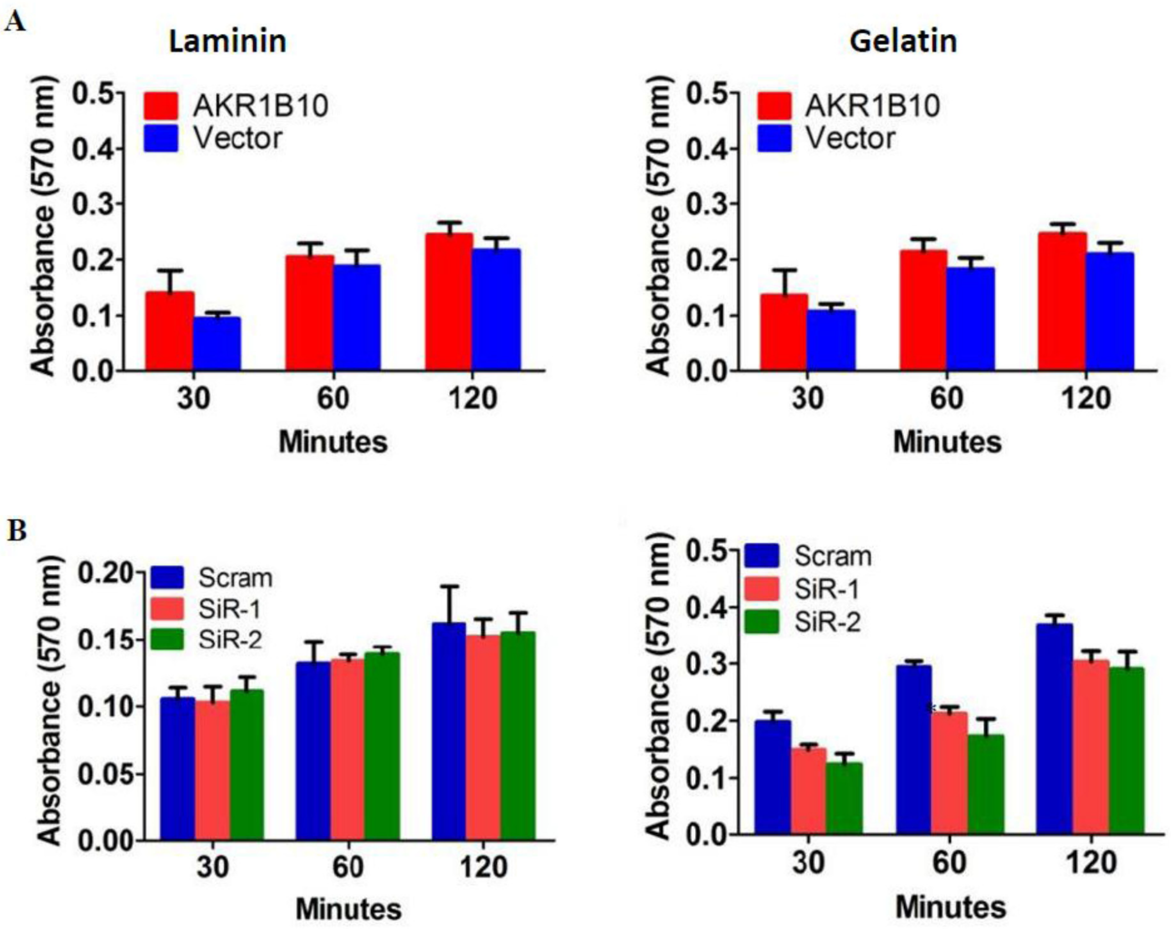

Supplementary Figure S2: No effects of AKR1B10 on breast cancer cell adhesion to laminin and gelatin. Breast cancer cells were separated with 2mM EDTA in PBS, spread in96-well plates coated with laminin or gelatin for 30, 60 and 120 minutes, and analyzed by a colorimetric assay after crystal violet staining. **A.** MCF-7 cells. **B.** BT-20 cells.

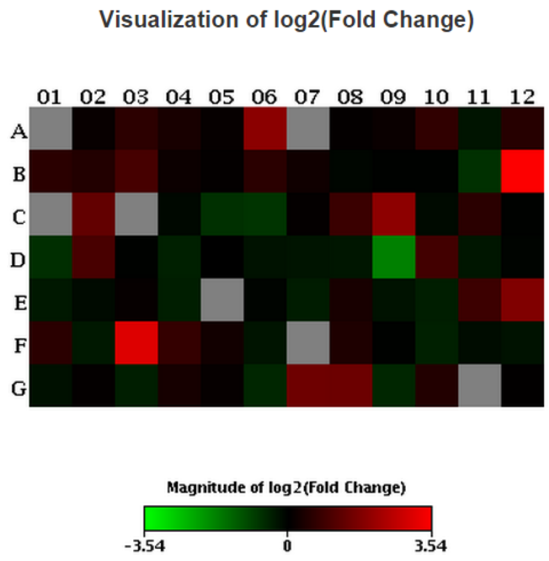

	1	2	3	4	5	6	7	8	9	10	11	12
A	ADAMTS1<br>-1.04	ADAMTS13<br>1.08	ADAMTS8<br>1.52	CD44<br>1.26	CDH1<br>1.05	CLEC3B<br>3.86	CNTN1<br>-1.04	COL11A1<br>1.04	COL12A1<br>1.11	COL14A1<br>1.59	COL15A1<br>-1.21	COL16A1<br>1.43
B	COL1A1<br>1.49	COL4A2<br>1.39	COL5A1<br>1.95	COL6A1<br>1.13	COL6A2<br>1.04	COL7A1<br>1.51	COL8A1<br>1.16	CTGF<br>-1.06	CTNNA1<br>-1.01	CTNNB1<br>-1.03	CTNND1<br>-1.60	CTNND2<br>11.63
C	ECM1<br>-1.04	FN1<br>2.55	HAS1<br>-1.04	ICAM1<br>-1.07	ITGA1<br>-1.59	ITGA2<br>-1.66	ITGA3<br>1.04	ITGA4<br>1.75	ITGA5<br>3.92	ITGA6<br>-1.10	ITGA7<br>1.48	ITGA8<br>-1.02
D	ITGAL<br>-1.57	ITGAM<br>2.00	ITGAV<br>-1.01	ITGB1<br>-1.37	ITGB2<br>1.00	ITGB3<br>-1.18	ITGB4<br>-1.22	ITGB5<br>-1.23	KAL1<br>-3.43	LAMA1<br>1.91	LAMA2<br>-1.23	LAMA3<br>-1.04
E	LAMB1<br>-1.26	LAMB3<br>-1.09	LAMC1<br>1.05	MMP1<br>-1.34	MMP10<br>-1.04	MMP11<br>-1.04	MMP12<br>-1.30	MMP13<br>1.26	MMP14<br>-1.19	MMP15<br>-1.34	MMP16<br>1.79	MMP2<br>3.46
F	MMP3<br>1.49	MMP7<br>-1.27	MMP8<br>8.51	MMP9<br>1.64	NCAM1<br>1.18	PECAM1<br>-1.22	SELE<br>-1.04	SELL<br>1.34	SELP<br>-1.03	SGCE<br>-1.37	SPARC<br>-1.12	SPG7<br>-1.20
G	SPP1<br>-1.16	TGFBI<br>1.04	THBS1<br>-1.34	THBS2<br>1.24	THBS3<br>1.05	TIMP1<br>-1.44	TIMP2<br>2.89	TIMP3<br>2.83	TNC<br>-1.43	VCAM1<br>1.39	VCAN<br>-1.04	VTN<br>1.02

Supplementary Figure S3: Expression of cell adhesion molecules (CAMs) in MCF-7 cells. CAMs mRNA levels were measured by a RT profiler PCR array, and data are expressed as fold changes of the CAMs in AKR1B10 expression MCF-7 cells over the vector control. *Upper panel*, heat map. *Lower panel*, a table for detailed information for each gene.

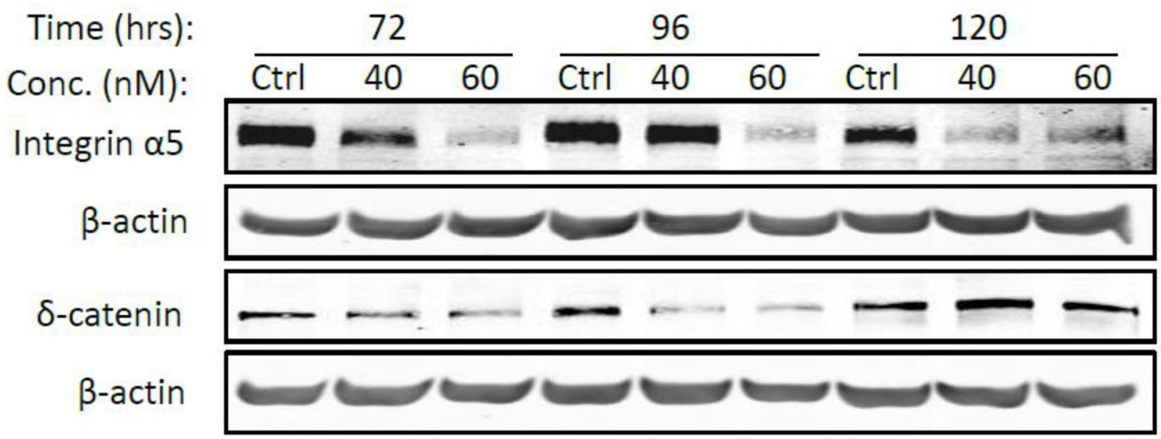

Supplementary Figure S4: SiRNA-mediated silencing of integrin α5 and δ-catenin genes in MCF-7 cells. MCF-7cells were transfected with an integrin α5siRNA pool (*upper*) or a δ-catenin siRNA pool (*lower*) at 40 or60nM, or with a scrambled siRNA pool at 60nM(Ctrl). Western blot was performedat 72, 96 and 120 hours post transfection. Conc, concentration.

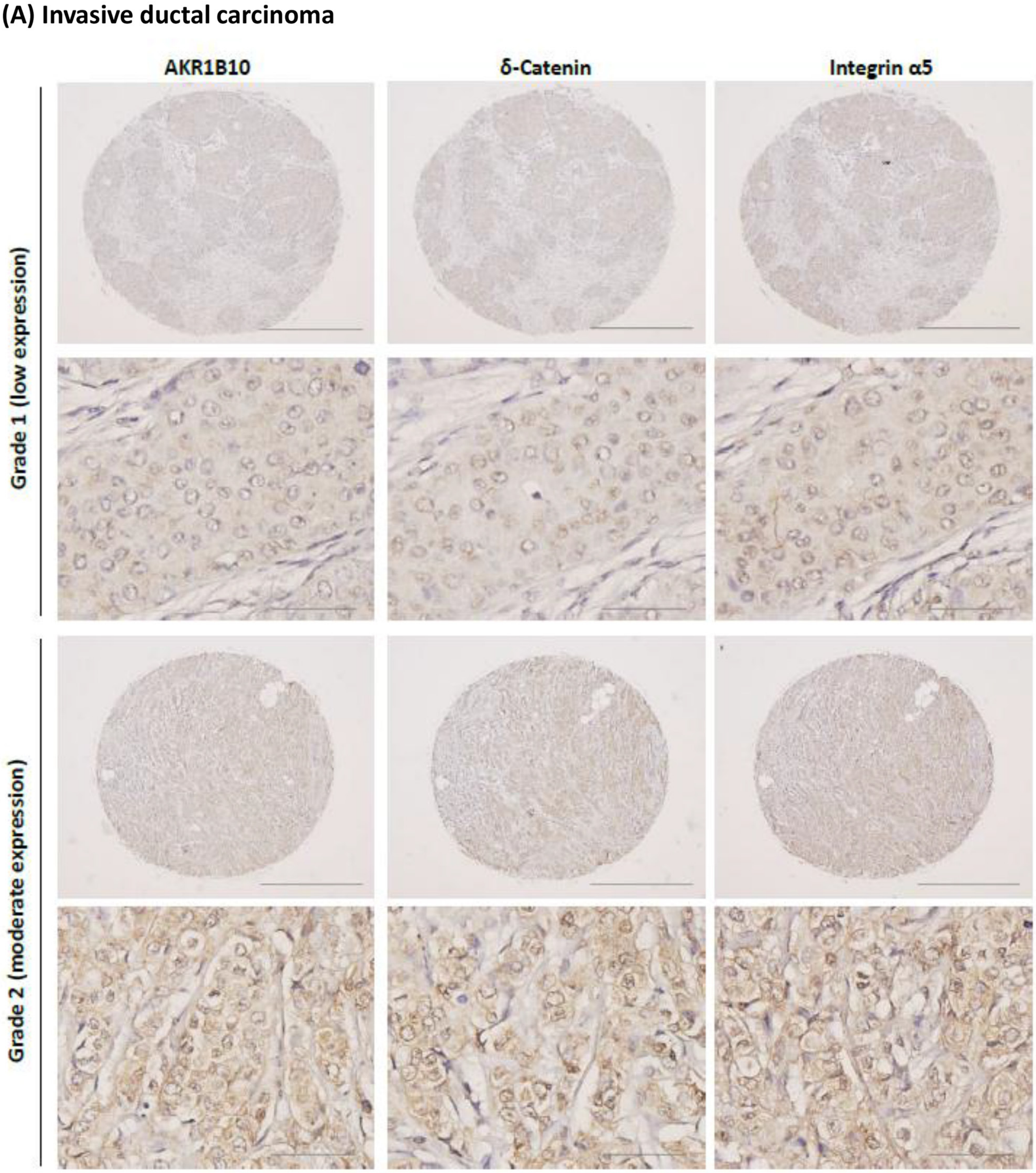

Supplementary Figure S5: AKR1B10, integrin α5,and δ-catenin expression in breast cancer tissues. The expression of AKR1B10, integrin α5 and δ-catenin in normal and malignant breast tissues was examined by immunohistochemistry as described in Material and Methods. **A.** Representatives of AKR1B10 expression at low or moderate levels in invasive ductal carcinomas. (*Continued*)

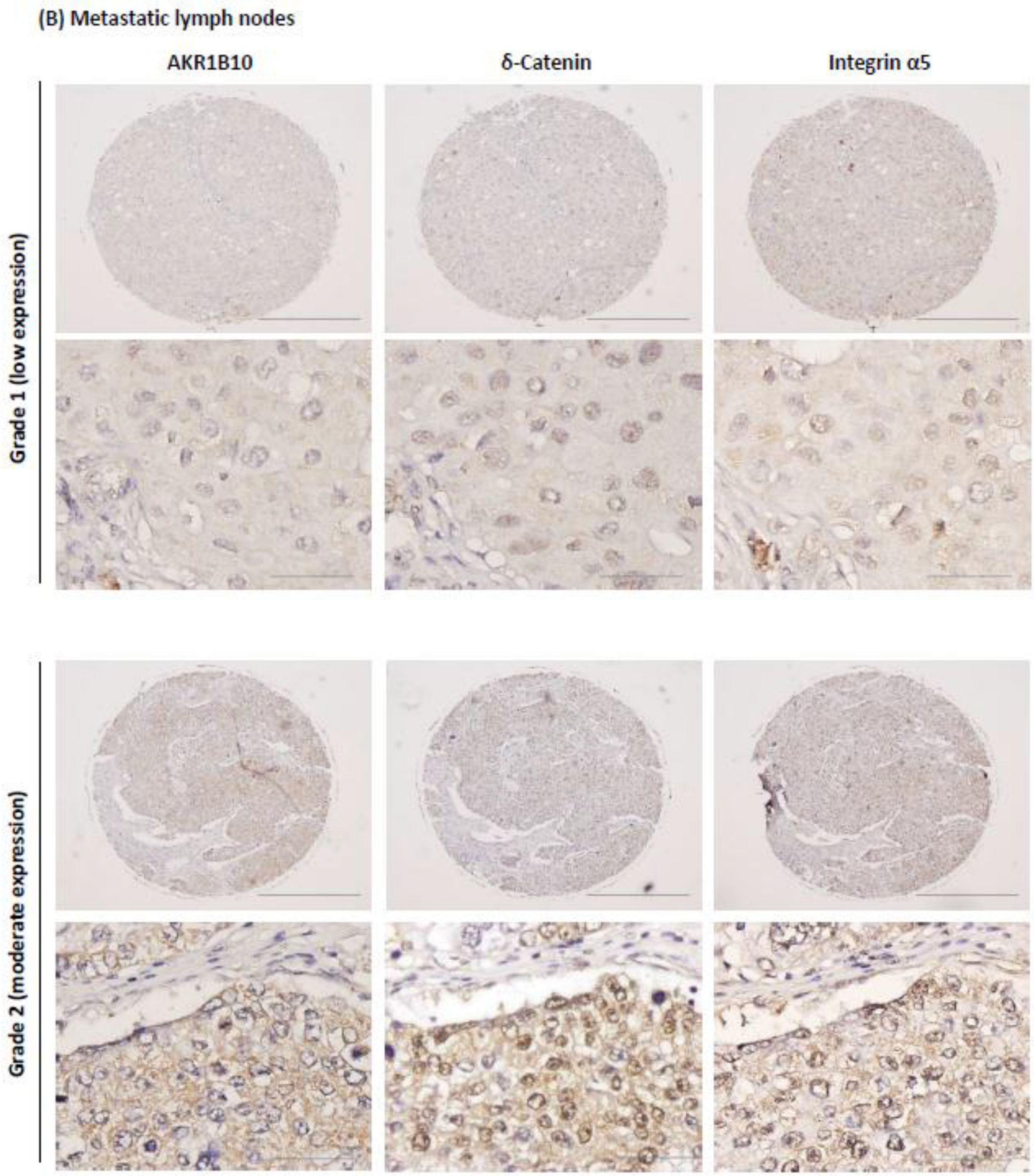

Supplementary Figure S5: (Continued) AKR1B10, integrin α5,and δ-catenin expression in breast cancer tissues. B. Representatives of AKR1B10 expression at low or moderate levels in metastatic lymph nodes. Scale bar = 50μM (40×) or 500μM (4×).

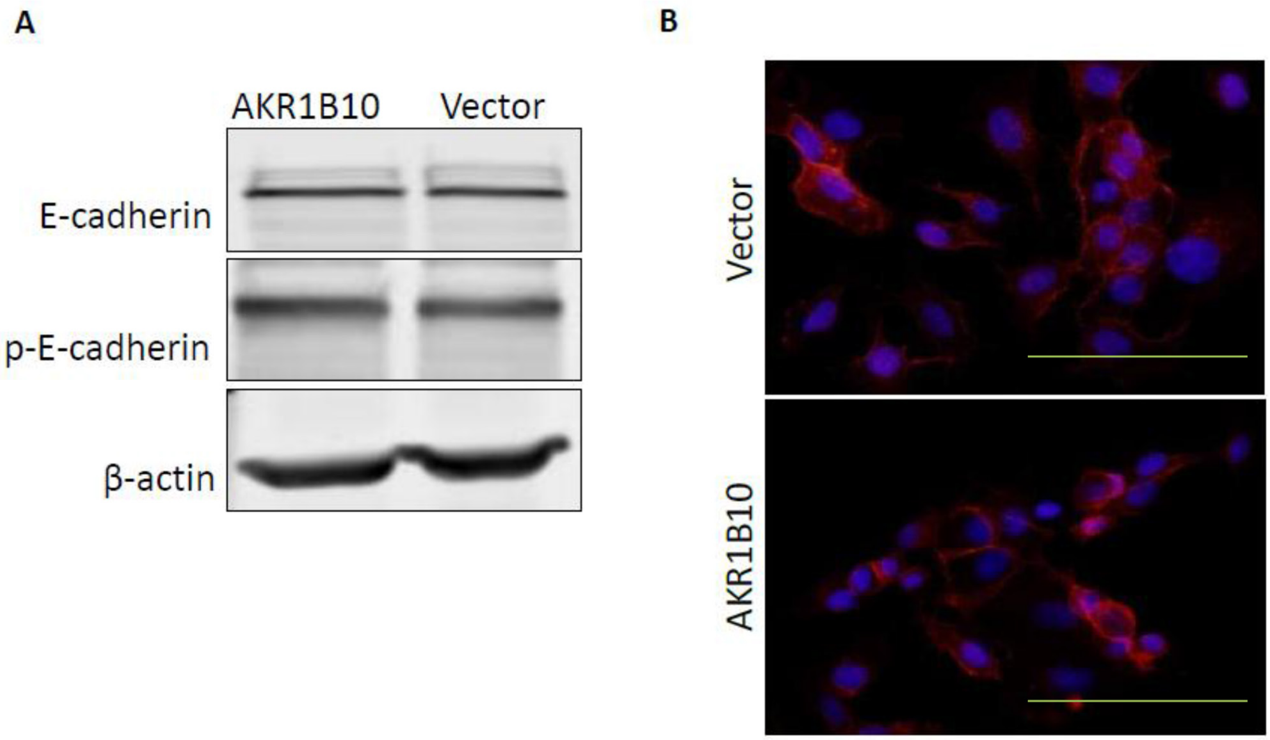

Supplementary Figure S6: E-cadherin and vimentin expression in MCF-7 cells. MCF-7 cells with ectopic expression of AKR1B10 or vector control were subjected to **A.** Western blot analyses for the expression of E-cadherin, and p-E-cadherin proteins, and to **B.** immunocytochemistry for the subcellular location of E-cadherin (Red: E-cadherin; Blue: DAPI for nuclei). AKR1B10 has not effects on expression, phosphorylation, and subcellular location of the E-cadherin. Scale bar= 200×.

Supplementary Table S1: Gene specific primer sequences for qRT-PCR

See Supplementary File 1
