# Supplementary figures and images for: AKR1B10 promotes breast cancer metastasis through integrin α5/δ-catenin mediated FAK/Src/Rac1 signaling pathway
Source: Oncotarget. 2016 May 27;7(28):43779–91. doi: 10.18632/oncotarget.9672 (PMC5190059; doi:10.18632/oncotarget.9672)

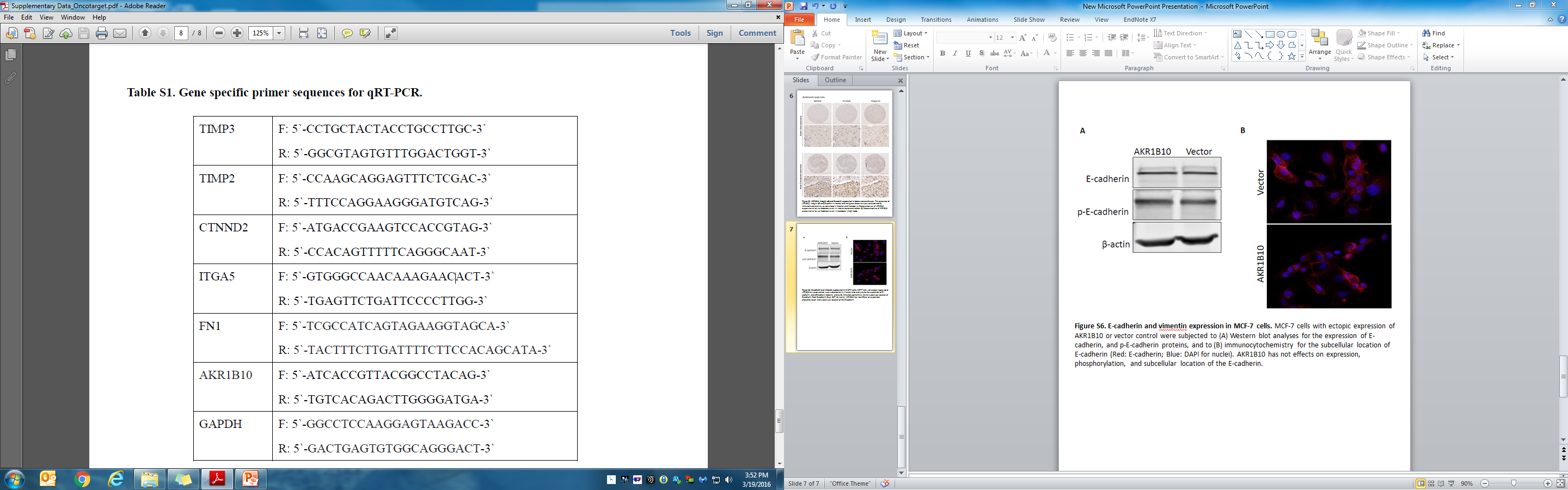

Supplement: Supplementary file 2 [file oncotarget-07-43779-s002.docx]
